# Community Based Assessment of Unintentional Injuries in a Community Development Block of Purba Bardhaman District, West

**DOI:** 10.4314/ejhs.v31i2.10

**Published:** 2021-03

**Authors:** Bengal Soumyaneel Das, Rabindra Nath Roy, Dilip Kumar Das, Amitava Chakraborty, Raston Mondal

**Affiliations:** 1 Department of Community Medicine, Burdwan Medical College, PurbaBardhaman, West Bengal, India

**Keywords:** Unintentional, Injuries, Household, Hazard

## Abstract

**Background:**

Injuries are a focus of public health practice because they pose a serious health threat and are preventable. Currently, injury accounts for 14% of all Disability Adjusted Life Years (DALYs) losses for the world's entire population. In India, unintentional injuries within the home environment have not so far been recognized to the same extent as traffic and work-related injuries among all age groups. With this background, a community based epidemiological study was conducted with the aim to find out the prevalence and epidemiology of unintentional injuries.

**Methods:**

A cross-sectional study was conducted during July 2018 - June 2019 in Bhatar block of Purba Bardhaman District. Cluster random sampling was applied to select required sample of 555 individuals from 24 villages. The study tools used were a predesigned and pretested schedule developed by the researchers with the help of Guidelines for conducting community surveys on injuries by World Health Organization (WHO) and a checklist for assessing household level injury hazard. The study had approval from Institutional Ethics Committee. Chi square test and multivariable logistic regression were performed using SPSS V16.

**Results:**

Prevalence of unintentional injury was 8.8 % in the preceding three months. Multivariable logistic regression revealed that those who were below 18 years of age, severely vulnerable to unintentional injuries and belonged to nuclear families had significantly higher odds of developing unintentional injuries at home.

**Conclusion:**

Unintentional injury is prevalent in West Bengal. Dissemination of injury prevention information with special focus on household modification is an effective strategy to prevent unintentional injuries.

## Introduction

Injury has been recognized as a major health problem in most high-income countries, especially in those countries that have experienced recent increase in industrialization and motorization, such as countries of East Asia and Latin America (1). It has been estimated by the World Health Organization (WHO) and Global Burden of Disease (GBD) study that unintentional injury accounts for 3.9 million deaths worldwide (2). Of these, 3.9 million deaths, about 90% occur in low and middle-income countries. The majority of these deaths are due to road traffic injuries, falls, drowning, poisoning and burns (2). Injury is one of the leading causes of adult mortality and a major contributor to disability in most age groups, even in the lower income countries such as those of South Asia and Africa (1,3,4). In spite of increasingly significant burden of death and disability, limited attention has been paid to injury as a health problem globally and more precisely in low-income countries.

Injury imposes health related burden to the society such as a product of the incidence and the duration of the treatment and rehabilitation period. The incidence of the fatal accidents or other events leading to fatal injuries is very much lower in comparison with the incidence of events which victims survive. Thus, the estimates of the impact on health that injuries impose on a given population are virtually impossible to derive when using only mortality statistics. One may also turn into the paradox when turning to programme evaluation, that better a fatality prevention programme occurs, poorer chance to prove its effect.

All injury events and deaths in a population are not captured by hospital-based injury surveillance systems. Household or community-based surveys are an important supplement to hospital-based surveillance, because they have the potential to gather more detailed information on both injury events and risk factors for injury. The policy makers also give low priority for injury related issues, and only few plans are drawn for injury prevention. Most of the studies are either hospital-based pertaining to injury pattern and prevalence among a particular age group. Community based study involving all age groups is rarely seen. In India, unintentional injuries within the home environment have not so far been recognized to the same extent as traffic and work-related injuries. With this background, a community based epidemiological study was conducted with objective to find out the prevalence and epidemiology of unintentional injuries at community level.

## Materials and Methods

A community based cross-sectional study was conducted from July 2018 - June 2019 in Bhatar block, randomly chosen from a total 23 community development blocks of Purba Bardhaman District, West Bengal. It consists of 14 Gram Panchayets and 107 villages. All individuals of all age groups who resided in the households of the study area at least for six months prior to the actual conduct of the study were considered as study subjects. Those who were not found even after two visits, not willing to participate in the study and found as guests or visitors in the households were excluded.

In spite of extensive PubMed search, no published literature was available regarding the prevalence of unintentional injury in Purba Bardhaman District or any other districts of West Bengal. However, in a study by Chowdhury SH, Karim MN, Rahman MR et al. (5) in the ‘Bairag’ Union of Anwara upazilla in Chittagong District of Bangladesh (having almost similar socio-cultural context as of India) the prevalence of unintentional injury was reported as 6.3%

Based on this data, considering 6.3% prevalence (p=0.063), 95% confidence interval, absolute error of 3% (L=0.03), design effect (f=2) and 10% drop outs; using the formula {Z^2^(p)(1-p)(f)(1.1)}/L^2^ the minimum effective sample size was 555 members of households. Considering average number of members in a household was four (as estimated by a pilot study), the total number of households required was (555/4 =138.75) i.e. 139. Households were the primary sampling unit.

According to “Guidelines for conducting community surveys on injuries and violence” by WHO (6), the cluster size usually corresponds to the number of households the researcher can cover in one day. Based on pilot study experience and other feasibility issues, it was estimated that the number of households that can be covered in one day is six. Thus, the cluster size was six households and the total number of required clusters (villages) was 139/6 = 24 (approx.). Therefore, from these 24 villages (clusters) a total of 144 households were included.

The required number of households was selected by cluster random sampling technique. Village was considered as cluster. Applying the principles of population proportion to size and methodologies of cluster sampling, 24 clusters were identified for the study out of 107 villages. In the second stage, 6 households were selected from each of these selected 24 clusters. At first, the location of center of each village was identified. Then, from this center point, any one of the lanes was selected randomly in any direction and six consecutive households were selected for data collection. Finally, all the members in the selected households fulfilling the eligibility criteria were included as study subjects.

Study tool used was a predesigned and pretested schedule developed by the researchers with the help of guidelines for conducting community surveys on injuries and violence by World Health Organization (6). A checklist was used for assessing household level injury hazard.

In the study, any unintentional injury requiring to seek medical attention (both from registered and non-registered medical practitioners) or to stay away from work or study or restrictions of activities of daily living for at least one day was recorded as injury event. All intentional injury cases, iatrogenic injuries including birth injuries, impairment and/or disabilities due to a disease other than injury or accident, and minor/trivial injuries i.e. an injury event when home remedies itself was sufficient and there was no absenteeism from school or work or there were no restrictions of activities of daily living were excluded from injury events.

Members were briefed about the purpose and nature of the study. Consent was also obtained before data collection. The study had approval from Institutional Ethics Committee. In the selected households, the information regarding household characteristics, demography, SES and other relevant information were collected from any senior member. The injury event was collected from individual members (victims) or from care giver in case of a child who were unable to give correct description of the injury event. Each episode of injury was recorded in a separate schedule. If the injury victim was absent at the time of interview, the head of the household or household member who knew most about the injury were interviewed as a proxy respondent. All cases of unintentional injuries occurring in the last three months preceding the date of interview were recorded. If available, relevant records were also reviewed and necessary information noted in the schedule.

A composite injury hazard score was calculated for all the study households taking into account all the household and environmental hazardous conditions in and around the house (within 500 meter of the household) like unsafe floor of living room, bathing place, cooking place, inadequate lighting in living room, bathing place, cooking place, unsafe cooking environment, dangerous/inflammable items stored in the household, naked electric wire/open switches, table fan on floor/low set ceiling fan, ponds/well/water bodies within 500 meter of house, heap of garbage/bush within 50 meter of house, domestic animals in house, unsafe climbing system and absence of protective guard on the margin of the roof giving equal weight-age to all the hazardous conditions. Unsafe floor of living room/bathing place/cooking place was operationally defined as presence of uneven floor or slippery/glaze tiles. Unsafe cooking environment has been defined as presence of movable burning Chula/LPG connection not conforming standard norms/pressure stove on the floor of the cooking place. Thus, total score was 15. Households were classified into three categories: mild (score 0–4), moderate (score 5–8) and severe (score 9–15) according to their vulnerability to unintentional injuries. Similar scoring system was used by Banerjee S et al in a community based cross-sectional study conducted in the rural area of Singur block, situated in Hooghly District of West Bengal, India (7).

Chi-square test and multivariable binary logistic regression were performed using Statistical Package for Social Sciences (SPSS) version 16.0 software. (SPSS, Inc, Chicago, IL, USA). P≤0.05 was considered statistically significant. Unintentional injury at home in the last three months was the dependent variable of binary multivariable logistic regression.

## Results

**Socio-demographic characteristics of the study subjects and different household and environmental characteristics**: Out of a total of 555 study subjects, 18–44 years constituted the maximum population with 53.7%. And, 0–17 years constituted 22.9% and ≥ 60 years constituted 19.8 %. On the other hand, 45–59 years constituted 3.6 % only. About 284(51.2 %) study subjects were males. Maximum study subjects were married (66.1%). The majority of them belonged to general caste (59.1%); scheduled tribe constituted 5.4% only. Out of the total 555 study subjects, the majority were “At home/retired” (25.2 %), followed by home maker (16.2%), agricultural laborer (15.1%) and student (13.9%). The maximum study subjects belonged to lower socio-economic status (39.1%). Only 2.2% of the study subjects belonged to upper socio-economic status as per the Modified BG Prasad Scale CPI (IW): 309 (March 2019).

There were a total of 144 households; of these, 82(56.9%) had kuccha houses and 24 (16.7%) had pucca houses. In maximum households, there was inadequate lighting in cooking place (60.4%). In 18 households (12.5%), there was provision to climb to the rooftop. Of these, 7(38.9%) had unsafe climbing system.

Based on the household and environmental hazard score mentioned earlier, the maximum households belonged to mild category (54.9%). Only 21.5% households belonged to severe category. When we did the analysis on individual basis, it was found that out of 555 individuals, the maximum (62.3%) were mildly vulnerable to unintentional injuries. Yet, 21.5% were moderately vulnerable and only 16.2% were severely vulnerable to unintentional injuries.

**Injury event factors and different epidemiological factors associated with unintentional injuries**: Out of the total 555 study subjects, 49 had history of unintentional injuries during the preceding three months from the actual date of data collection. All the 49 study subjects had single episode of injury. Thus, the total number of injury episodes was 49. Therefore, the prevalence of unintentional injury in the last three months was 8.8%.

**Figure 1:**
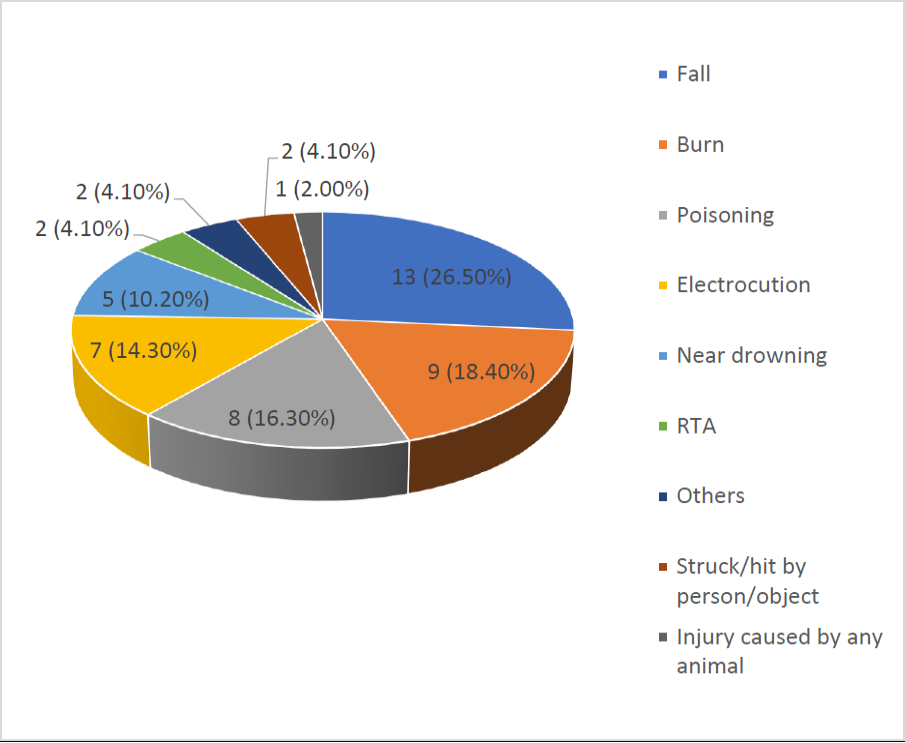
Pie diagram showing distribution of injury episodes according to cause of injury (n = 49).

Distribution of the injury episodes according to cause of injury has been shown in Figure 1. Out of a total of 49 injury episodes, 13(26.5%) were fall-related injury. Only 1(2.0%) injury episode was caused by animal (dog bite). “Others” includes broken glass injury and foreign body injury eye. Out of a total of 13 fall-related injuries maximum was on the ground level (46.1%). Fall from stairs constituted 38.5% of all the fall-related injuries. Contact with hot object or solid substance constitute 44.5% of all the burn injuries. There was a total of five near drowning injuries in the last three months. Out of these five episodes, near drowning in pond at/within 500 meter of the house constituted 80%. There was a total of eight poisoning injuries in the last three months. Out of these eight episodes, 4(50%) occurred due to drug or medical substance used mistakenly or in overdose.

**Figure 2:**
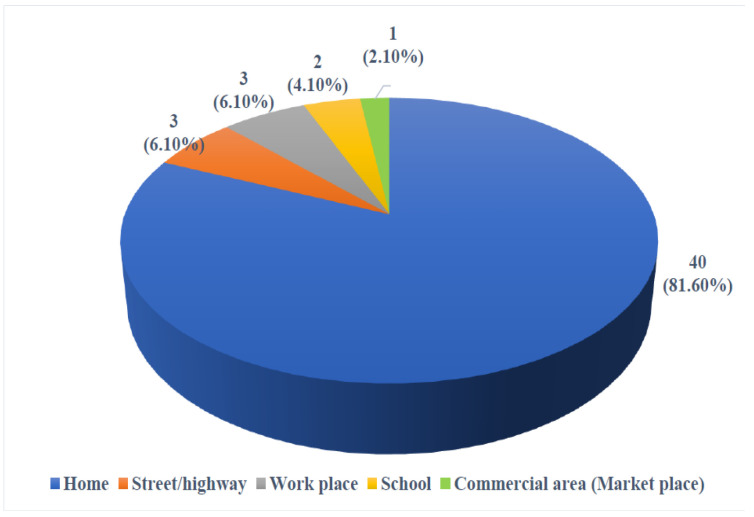
Pie diagram showing distribution of injury episodes according to place of occurrence (n = 49).

The distribution of injury episodes according to place of occurrence is shown in Figure 2. Out of a total of 49 injury episodes, the maximum i.e. 40(81.6%) occurred at home. Out of a total of 49 injury episodes, 19(38.8%) episodes occurred during leisure activities. Fracture occurred in 12.2% of the injury episodes. In 20.4% of the injury episodes, there was cut or other open wound. In 12.2% of the injury episodes, there was bruise or superficial injury. In only 4.1% of the injury episodes, there was concussion or head injury. Lower limb was the site of injury in the maximum number of cases (32.6%) followed by upper limb (28.6%). Most of the injury episodes (53.1%) occurred during 6 AM to 12 PM.

Unintentional injury in the last three months was significantly associated with age group, marital status, religion, occupational status and type of family as evident from significant result of Chi-square test. However, it was not significantly associated with sex, educational status, socioeconomic status and caste. The association between unintentional injuries at home and different variables is described in Table 1.

**Table 1:** Association between unintentional injury at home and different variables (n = 555)

Socio-demographic and other variables	Total number	Unintentional injury at home		Chi square value, Df and ‘p’ value

		Yes (%)	No (%)	
**Age (in years)**				
0–17	127	20 (15.7)	107 (84.3)	19.027, Df=3
18–44	299	13 (4.3)	286 (95.7)	p < 0.05*
45–59	20	3 (15.0)	17 (85.0)	
≥ 60	109	4 (3.7)	105 (96.3)	
**Sex**				
Male	284	20 (7.0)	264 (93.0)	0.024, Df=1
Female	271	20 (7.4)	251 (92.6)	p =0.878
**Religion**				
Hindu	410	38 (9.3)	372 (90.7)	9.968, Df=1
Muslim	145	2 (1.4)	143 (98.6)	p = 0.002
**Caste**				
General	328	27 (8.2)	301 (91.8)	1.259, Df=1
SC/ST/OBC	69	12 (17.4)	57 (82.6)	p = 0.262
**Type of family**				
Nuclear	194	36 (18.6)	158 (81.4)	57.446, Df=1
Joint	361	4 (1.1)	357 (98.9)	p < 0.05
**Marital status**				
Married	367	13 (3.5)	354 (96.5)	24.709, Df=2
Unmarried	134	22 (16.4)	112 (83.6)	p < 0.05
Separated/widowed	54	5 (9.3)	49 (90.7)	
**Occupational status**				
Unemployed/at home/student/home-maker	325	31 (9.5)	294 (90.5)	6.373, Df=1
Business/working outside the house/others	230	9 (3.9)	221 (96.1)	p = 0.012
**Socio-economic status**				
Upper/Upper-middle/Middle/Lower-	338	34 (10.1)	304 (89.9)	10.514, Df=1
middle				p = 0.001
Lower	217	6 (2.8)	211 (97.2)	
**Hazard**†				
Mild	346	1 (0.3)	345 (99.7)	89.003, Df=2
Moderate	119	18 (15.1)	101 (84.9)	p < 0.05
Severe	90	30 (33.3)	60 (66.7)	

* Fisher's Exact test has been done

† Household and environmental hazard

All the variables which were found to be significantly associated with unintentional injuries at home in last three months (Table 1) in Chi-square test were not put into analysis in the final multivariable logistic regression model. Most of the unintentional injuries at home have occurred in the unmarried (16.4%), and 94.8 % of the unmarried population were in the age group of below 18 years. Again, most of the injuries occurred in the age group of below 18 years. Thus, while doing regression, marital status was not kept with age group as a covariate due to redundancy. Thus, in the final model age group, sex, religion, type of family, occupational status, socio-economic status and household and environmental hazard were put into analysis (Table 2). The model was significant as evident from the omnibus Chi-square statistics (Chi square value = 81.091, p < 0.05). This model was a good fit as evidenced from non-significant Hosmer-Lemeshow statistics (p = 0.793). All independent variables could explain between 13.6% to 33.6% variance of the dependent variable using Cox and Snell and Nagelkerke R^2^. Overall, our model correctly predicted 93.5% of outcomes as shown by the classification table. Age group, type of family and household and environmental hazard were found to be significantly associated with unintentional injury at home in the last three months. Those who were below 18 years old belonged to nuclear families and severely vulnerable to unintentional injuries had significantly higher odds of developing unintentional injuries at home.

**Table 2:** Bivariate analysis and multivariable logistic regression for predictors of unintentional injury at home (n = 555)

Predictors of unintentional injury at home	OR (95 % CI)	AOR (95 % CI)
**Age (existences)**		
≥18	1 (ref)	1 (ref)
<18	3.813(1.980–7.343)*	2.784(1.026–7.553)*
**Sex**		
Male	1 (ref)	1 (ref)
Female	1.052(0.553–2.002)	1.342(0.557–3.232)
**Religion**		
**Muslim**	1 (ref)	1 (ref)
**Hindu**	7.304(1.739–30.671)*	1.788(0.360–8.888)
**Type of family**		
Joint	1 (ref)	1 (ref)
Nuclear	20.335(7.117–58.102)*	6.259(1.844–21.253)*
**Occupational status**		
Business/working outside the house/others	1 (ref)	1 (ref)
Unemployed/at home/student/home-maker	2.589(1.208–5.549)*	1.220(0.380–3.913)
**Socio-economic status**		
Lower	1 (ref)	1 (ref)
Upper/Upper-middle/Middle/Lower-middle	3.933(1.622–9.534)*	2.083(0.771–5.627)
**Household and environmental**		
**Hazard**		
Mild/moderate	1 (ref)	1 (ref)
Severe	11.538(5.782–23.026)*	3.975(1.761–8.969)*

* Statistically significant

## Discussion

Injury as a research problem has been largely ignored in developing countries. Rural and urban development in transition exposes households to unsafe environments. With advances in modern medicine and control of infectious diseases in the middle of this century, accidents have emerged as the principal threat to the health and welfare of people (8).

Injury prevention intervention requires precise estimation of burden of injuries at community level. Prevalence of unintentional injury in the last three months was 8.8% in the present study. Similar findings were reported by different studies. The recall period in the present study was three months. The recall period was different in different studies. In most of the studies, it was taken as one year. Therefore, the prevalence obtained in those studies may not be comparable with the prevalence in the present study.

In a study in villages in the state of Andhra Pradesh, India, by Cardona M A et al, (9) non-fatal injury was reported by 6.7% of survey participants. In a study in Bangladesh by Chowdhury SH et al. (5) crude prevalence of injury over last one year was calculated to be 6.3% (95% CI 5.69%–6.89%).

It was found in our studies that out of a total of 49 injury episodes, the maximum was fall injuries (26.5 %). Fall constituted 34.7% of all the injuries in a study done by Paul B et al.; of which 38.6% occurred inside the victims' homes (10). In our study, burn, poisoning, electrocution and near drowning constituted 18.4%, 16.3%, 14.3% and 10.2% respectively. Only 1(2.1 %) injury episode was caused by any animal (dog bite injury). In a community based cross-sectional epidemiological study in a rural community in Bangladesh by Chowdhury S et al. (5), it was found that the mechanisms of injury of 30% subjects were road accident. Mechanism of injury of 29.5% subjects was slip, trip or fall. Around 10% had deep cut, 1.4% had superficial cut, and 3.3% had other injuries including burn. Among others, 1.7% suffered electrocution, 0.7% were drowned, 0.9% were attacked by animal and 1.2% were bitten by animal or stung by insects. The results of the present study were also quite similar with that of the Andhra Pradesh study by Cardona M A et al (9). Fall was found to be the most common cause of non-fatal injuries (38%).

Lower limb was affected in the maximum injury episodes (32.6 %) followed by upper limb (28.6%). In a community based cross-sectional epidemiological study of injury in a rural community in Bangladesh by Chowdhury SH et al.(5) it was found that both the extremities together constituted more than 70% of the reported injury. The study in Aligarh by Zaidi S et al. (11) showed that the most common primary body parts affected by injury were lower limbs (39.6 %) followed by head, face and neck (31.9%), and the least to chest and abdomen (1.2%).

In the present study, it was found that those who were severely vulnerable to unintentional injuries were having significantly higher odds of developing unintentional injuries at home even when adjusted for other variables [AOR 3.975 (1.761 – 8.969)]. A community based cross-sectional study conducted in the rural area of Singur block, situated in Hooghly District of West Bengal, by Banerjee S et al. (7) revealed similar findings. Household level injury hazards were found to be significantly associated with injury occurrence even when adjusted with other variables [AOR 1.55 (1.3–1.8)]. Híjar-Medina et al. also mentioned that unprotected electric circuits, lack of protection rails to staircases, and free access to the roof were potential risk factors for unintentional home injuries in children <10 years of age (12).

One of the important limitations of this study is rooted in its reliance on self-reporting by respondents. The accuracy of respondents' answers on the occurrence of injury events can not be independently verified. There is tendency among respondents to report events occurring outside the recall period as if they had occurred within it. The accuracy of recall is also influenced by memory decay. There is high chance of recall bias particularly for less serious injuries. The use of proxy respondents in the absence of first choice respondents tends to lead to underreporting of injury events. In addition, if a respondent is answering on behalf of more than one individual, memory decay might be more extensive.

Unintentional injuries are a form of significant morbidity in the community level and need to be addressed urgently. Dissemination of injury prevention information with special focus on household modification is an effective strategy to prevent unintentional injuries.

## References

[1] Smith GS, Barss P (1991). Unintentional injuries in developing countries: the epidemiology of a neglected problem. Epidemiological Reviews.

[2] World Health Organization (2008). The Global Burden of Disease: 2004 update.

[3] Murray CJ, Lopez A (1996). The global burden of disease, vol.1: *a comprehensive assessment of mortality and disability from diseases, injuries and risk factors in 1990 and projected to 2020*.

[4] Feachem RG (1992). The health of adults in the developing world.

[5] Chowdhury SH, Karim MN, Rahman MR, FAiz MA, Ahmed F, Selim S (2015). An epidemiological study of injury in a rural community in Bangladesh. Bangladesh Med Res Counc Bull.

[6] WHO Guidelines for conducting community surveys on injuries and violence.

[7] Banerjee S, Paul B, Bandyopadhyay K, Dasgupta A (2016). Domestic unintentional injury of 1 to 5-year-old children in a rural area of West Bengal, India: a community-based study. Tanzania Journal of Health Research.

[8] Scheidt PC, Jones DH (1995). The Epidemiology of non fatal injuries among US children and youth. Am J Pb H.

[9] Cardona M, Joshi R, lyengar S, Ramkrishna G, Dandona R, Stevenson MR, Neal BC (2008). The burden of fatal and non-fatal injury in rural India. Inj Prev.

[10] Paul B, Sinha D, Misra R, Basu M, Ray S (2017). Physical injury: Is it inevitable or preventable? an experience from a Tertiary Care Hospital of Kolkata, West Bengal. Medical Journal of Dr. DY Patil University.

[11] Zaidi SH, Khan Z, Khalique N (2013). Injury pattern in children: a population-based study. Indian journal of community health.

[12] Híjar-Medina MC, Tapia-Yáñez JR, Lozano-Ascencio R, López-López MV (1992). Home accidents in children less than 10 years of age: Causes and consequences. Salud Publica Mex.

